# Deep Brain Stimulation of the VIM for Tremor in a Patient with POLR3A-Associated Cerebellar Syndrome Without Long-Term Benefit

**DOI:** 10.5334/tohm.1003

**Published:** 2025-04-16

**Authors:** Ute Scheller, Steffen Paschen, Fabian Maass, Christoph van Riesen

**Affiliations:** 1 University Medical Center Göttingen, Department of Neurology, Göttingen, Germany; 2 Department of Neurology, University Hospital Schleswig-Holstein, Campus Kiel Germany; 3 Christian Albrechts-University of Kiel, Kiel, Germany; 4 German Center for Neurodegenerative Diseases (DZNE), Göttingen, Germany

**Keywords:** POLR3A, deep brain stimulation, tremor

## Abstract

**Background:**

Deep brain stimulation is an approved therapy for essential tremor and Parkinson’s disease. In addition, VIM-DBS is used off-label for the treatment of tremor syndromes with a different etiology.

**Case Report:**

We present the case of a woman with a drug-refractory action tremor due to rare compound heterozygous POLR3A mutations. Her treatment with VIM DBS did not lead to a sustained improvement of symptoms.

**Discussion:**

Tremor due to POLR3A-related cerebellar syndromes may not be responsive to VIM DBS. The networks contributing to cerebellar tremor should be better investigated in terms of where neuromodulatory therapy might be more effective.

VIM-DBS is an established treatment option for tremor due to essential tremor (ET) and Parkinson’s disease (PD) tremor, with long-lasting effects, although there may be some reduction in efficacy over time in ET [1]. In this case report, we present a patient with a predominantly cerebellar syndrome due to compound heterozygous variants in POL3RA, who received bilateral deep brain stimulation (DBS) of the ventral intermediate nucleus of the thalamus (Vim) for tremor treatment. Biallelic variants in POLR3A have recently been identified as a common cause of sporadic and recessive ataxia and spastic paraparesis [2,3,4,5] and treatment options remain scarce [6].

Onset was reported at 8–10 years of age with mild right limb action tremor, dystonic postures of the upper extremities and incoordination. At the age of 19, a small amplitude postural and kinetic tremor of both arms was noted in association with an ataxic gait and mild dysarthria. Gait ataxia and action tremor progressed over the following years, leading to severe impairment of daily activities by the age of 28 (Video 1). The extended family tree was free of neurological disease and the parents were not consanguineous.

**Video 1:** Patient examination at presentation.

At the time of examination (Video 1), smooth pursuit of eye movements was saccadic, and saccades were hypometric in all directions. Mild cerebellar dysarthria was present. The patient had rest, postural, kinetic and intention tremor of the upper limbs. Mild rest tremor was present in the upper right limb only. Mild to moderate postural tremor was present in both upper limbs. Kinetic and intention tremor was severe in both upper limbs and the main cause of disability. There was some dystonic posturing of both upper extremities. Head and lower extremities were not affected by the tremor. Functional tremor was excluded by clinical testing (no significant variability over time, no entrainment, no influence of mental distraction). Writing was extremely impaired. Tremor had worsened during the disease duration. The SARA Score was 17/40.

Additionally, gait was ataxic with an undirected tendency to fall. When walking, she exhibited dystonic postures of the right hand and foot. Head position was slightly dystonic. The patient presented with hypodontia, a typical symptom of the POLR3A spectrum, and required replacement with dental implants. A comprehensive cognitive examination revealed mild impairment of executive functions and reduced processing speed. MRI imaging showed mild cerebellar atrophy without evidence of hyperintensity along the cerebellar peduncles or the midbrain (Figure 1a–d). MRI of the cervical spine showed no abnormalities. Sensory and motor evoked potentials were delayed, suggesting central dysfunction. Whole exome sequencing revealed compound heterozygous variants in the POLR3A gene c.1909 + 22G>A (previously described as pathogenic) and c.1631A>C (predicted in silico to be pathogenic). The phenotype of our patient is consistent with previous descriptions of POLR3A-associated disease in the literature [2,3,4,5]. Common symptoms described include gait ataxia, upper limb tremor, spasticity, dysarthria, dental abnormalities and sometimes mild cognitive or gonadal dysfunction [2,3,4,5]. The majority of cases showed bilateral hyperintensities along the superior cerebellar peduncles on MRI, in addition to cerebellar atrophy and corpus callosum hypoplasia [2,3,4,5]. In contrast, in our case, hyperintensities along the cerebellar peduncles were absent and only mild cerebellar atrophy was seen, which did not progress significantly over time (Figure 1a–d).

**Figure 1:**
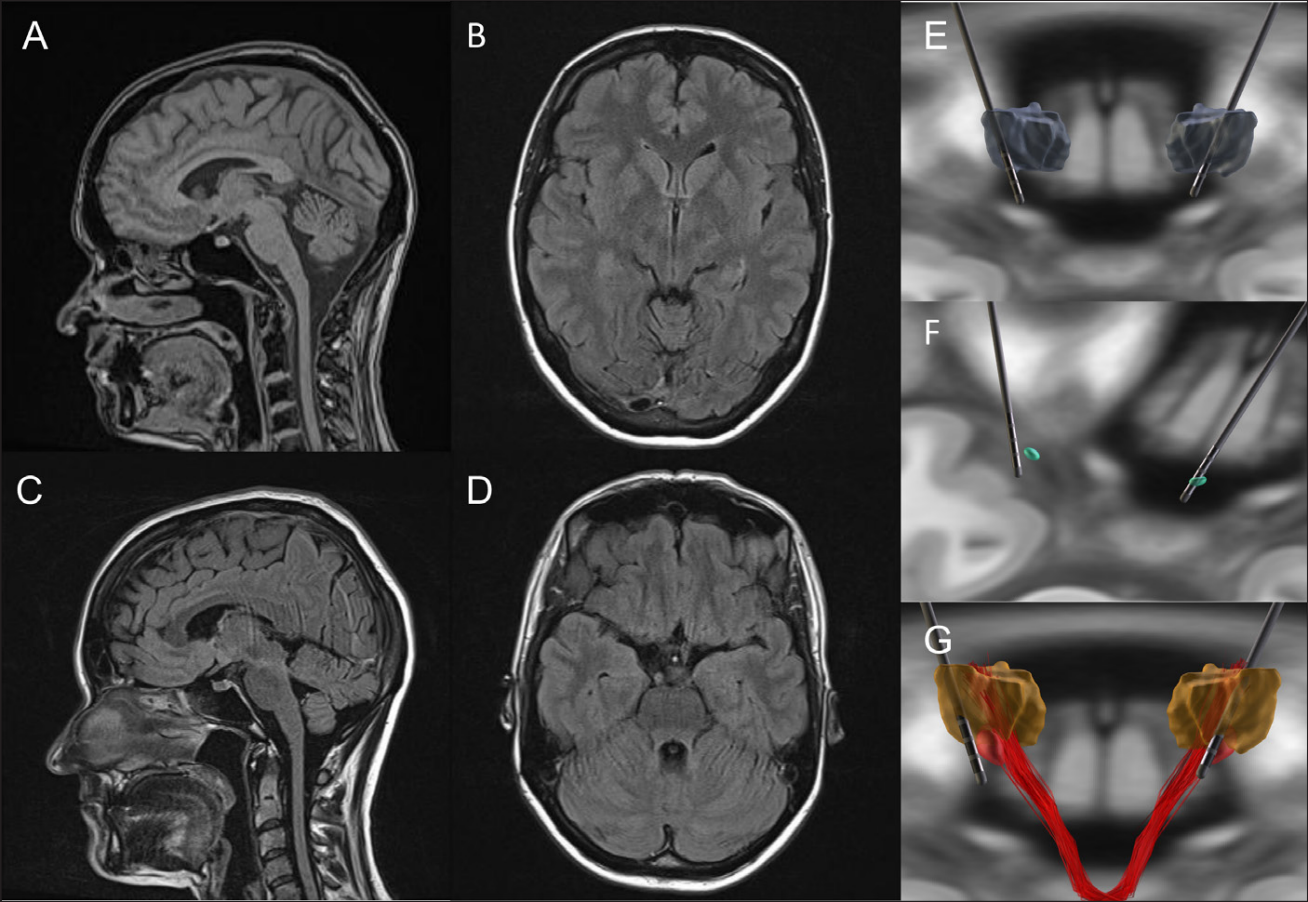
**MRIs and visualization of electrode placement.** MRIs **(A–D)** after DBS lead explantation and DBS electrode localization **(E–G)** calculated using Lead-DBS software8. E: Electrodes in relation to the Vim nucleus. F: Electrodes in relation to the published “sweet spot” for DBS in ET. G: Electrodes in relation to the Vim and the dentatorubrothalamic tract.

As in the other published cases, treatment proved difficult. Medications including beta blockers (propranolol, metoprolol), primidone, carbamazepine, gabapentin, topiramate, and botulinum toxin did not provide sufficient symptom relief or were not tolerated. The potential risks and benefits of DBS surgery were discussed in detail with the patient, emphasizing that a positive clinical outcome could not be predicted with certainty for her condition, which had not been identified at that time. Due to the severity of her functional impairment in daily life and the potential for a significant improvement in her quality of life, the patient agreed to the surgery. Therefore in 2011, DBS electrodes were implanted bilaterally in the VIM nucleus to control the tremor. A visualization of electrode placement and stimulation sites (Figure 1e–g) showed favorable and only slighty suboptimal electrode placement on the right and left, respectively. On the Fahn-Tolsa-Marin clinical tremor rating scale A/B/C the patient had a preoperative score of 18/21/13. One year postoperatively the score had changed to ON DBS of 23/32/16 and OFF DBS of 29/35/23. At the last visit in 2023 she had a score of 21/28/16 (Video 2). After 10 years of stimulation, the entire DBS system had to be explanted due to infection and was not subsequently replaced, because the patient did not notice any difference in tremor control after the explantation. The patient reported some reduction in tremor initially after surgery, which was soon lost thereafter. In the outpatient clinic the two lowest contacts were generally reported to have a small effect on tremor intensity in the first months after surgery but not thereafter despite little or no disease-related progression in tremor severity during this period and frequent reprogramming and switching off of the device at night (Video 2). Many different settings of DBS were tried, including monopolar, double monopolar and bipolar electrode configurations with different amplitudes (up to 3.5 V) and different frequencies (130–160 Hz) at a pulse width of 90 µs.

**Video 2:** Tremor examination of the patient before DBS, and after 1, 4, and 12 years.

However, good tremor control was never achieved, and the patient noticed side effects like aggravated gait impairment and paresthesia of the right upper limb. Additionally, the tremor increased significantly over time due to disease progression.

To date, there are only two published cases of patients with a POLR3A-related disorder that were treated with DBS [7]. A recently published patient showed a sustained, but very moderate benefit from VIM-DBS over a period of 5 years [8]. The achieved tremor suppression on the Tremor Research Group Essential Tremor Rating Scale (TETRAS) was 16%, which is much lower than the average suppression of 60% that has been reported for essential tremor in a meta-analysis [9]. In contrast to our patient who had mainly intention tremor, he had mainly postural and kinetic tremor. Another interesting difference, which may be related to the presentation of the tremor and its responsiveness to DBS is that, in contrast to our patient, the patient published by Minnerop et al had bilateral T2 hyperintensities of the superior cerebellar peduncles (SCP) through which the dentato-rubro-thalamic tract passes. The second published patient had parkinsonism and severe levodopa-induced dyskinesia, which were reduced by GPi DBS. In conclusion, in our patient with POLR3A-related cerebellar syndrome, Vim DBS did not result in a long-term benefit in tremor control, despite relative stability of tremor over more time. It remains to be determined, whether this unsatisfactory response to DBS is related to the preponderance of intention tremor as the main cause of disability and its unresponsiveness to neuromodulatory therapies in general or whether it is related to the pathophysiology of the POLR3A-related disease. Alternatively, the network involved in the generation of the tremor in POLR3A-related disorders might be significantly different from that involved in ET and PD, for which the electrode placement has been optimized. Alternative anatomical targets for which DBS treatment of tremor has been studied include the posterior subthalamic area, the zona incerta, the ventral oralis anterior and posterior thalamus (Voa, Vop), the globus pallidus, the superior cerebellar peduncle, and the dentate nucleus of the cerebellum [10,11]. Current evidence solely relies on case reports of patients with different disease etiologies and disease stages [10,11]. In the context of inherited degenerative ataxias, VIM-DBS has been shown to suppress tremor in patients with SCA-2, SCA-6, SCA-31, FXTAS and other unidentified genetic cerebellar syndromes [12]. The small number of patients treated per condition and the variable effect of treatment do not allow a definitive answer to be given about the efficacy of VIM DBS in degenerative cerebellar ataxias. The pathophysiology causing tremor may be very different even in this group of ataxias and possibly even within POLR3A-related diseases. It is also very likely that non-positive patient outcomes are under-represented in the literature due to the file drawer effect. Further systematic trials and registries of patients treated with DBS are needed to identify potential and specific targets for DBS in hereditary ataxias.
